# Corrosion Resistance of GMAW Duplex Stainless Steels Welds

**DOI:** 10.3390/ma16051847

**Published:** 2023-02-23

**Authors:** Argelia Fabiola Miranda-Pérez, Bryan Ramiro Rodríguez-Vargas, Irene Calliari, Luca Pezzato

**Affiliations:** 1 Department of Strategic Planning and Technology Management, Universidad Popular Autónoma del Estado de Puebla, 17 Sur, 901, Barrio de Santiago, Puebla 72410, Mexico; 2 Corporación Mexicana de Investigación en Materiales S.A. de C.V., Ciencia y Tecnología No. 790, Saltillo 400, Saltillo 25290, Mexico; 3 Department of Engineering, University of Perugia, Via G. Duranti 93, 06125 Perugia, Italy; 4 Department of Industrial Engineering (DII), University of Padua, Via Marzolo 9, 35131 Padua, Italy

**Keywords:** duplex stainless steels, robotic GMAW, corrosion, cracking, pitting

## Abstract

The hydrocarbon industry constantly requires a better understanding of stainless-steel welding metallurgy. Despite the fact that gas metal arc welding (GMAW) is one of the most commonly employed welding processes in the petrochemical industry, the process is characterized by the presence of a high number of variables to control in order to obtain components that are dimensionally repeatable and satisfy the functional requirements. In particular, corrosion is still a phenomenon that highly affects the performance of the exposed materials, and special attention must be paid when welding is applied. In this study, the real operating conditions of petrochemical industry were reproduced through an accelerated test in a corrosion reactor at 70 °C for 600 h, exposing robotic GMAW samples free of defects with suitable geometry. The results show that, even if duplex stainless steels are characterized for being more corrosion resistant than other stainless steels, under these conditions it was possible to identify microstructural damage. In detail was found that the corrosion properties were strongly related to the heat input during welding and that the best corrosion properties can be obtained with the higher heat input.

## 1. Introduction

New oil reserves are one of the more hostile environments for metallic materials from the point of view of corrosion. Venezuela, Saudi Arabia, and Canada are the leading countries in this production field, with Mexico currently situated in the 17th position, meaning that all infrastructure must be renewed, maintained, and preserved since detrimental components in petroleum are present [1,2,3,4]. Considering that corrosion is one of the most important issues in the oil and gas industry, a high demand for corrosion-resistant steels is always required. 

Stainless steels are the most suitable for these applications, with the martensitic and duplex ones most commonly employed in the oil and gas industry, where hydrogen sulfide is the most dangerous compound on the exterior platform [4,5]. 

Duplex stainless steels (DSS) are not resistant to the presence of Cl ions. In the same way, under certain temperature conditions and concentrations of S, stainless steels decrease their anticorrosion response and initiate their mechanical failure [5,6]. The petrochemical industry has a growing interest in this type of material because it provides benefits and savings, not only in better performance of the components but also in hidden savings derived from maintenance reduction, thus increasing industrial efficiency. In a study performed by Hruska et al. for the biomass industry, stainless steels were coated, resulting in improved corrosion properties [7]. Stainless steels are a group of high-alloy steels based on the Fe-Cr, Fe-Cr-C, and Fe-Cr-Ni systems. To be considered stainless, they must have a minimum Cr content of 10.5%. These types of steels are a large group of special alloys developed primarily to resist to corrosion phenomena [5]. Other relevant characteristics of these materials include excellent formability, resistance to high and cryogenic temperatures, and resistance to oxidation and cracking at high temperatures [8]. In the oil and gas industry, welding is widely employed, and the selected process must guarantee the performance of the components and should not require excessive maintenance in order to achieve high industrial efficiency.

It is well known that poor practices during the welding process of stainless steels result in a significant reduction in corrosion resistance, particularly in stress corrosion cracking (SCC) resistance. Even though DSS are known to be very resistant to classical sensitization due to chromium carbide precipitation, currently, the sensitization phenomenon in DSS can occur due to the precipitation of intermetallic compounds such as σ (sigma) or χ (chi) and other particles or phases such as Cr_2_N (chromium nitrides) or α’ (alpha prime) [9,10]. The formation of σ is by far the factor with the greatest impact on reducing the resistance to SCC in this type of material; however, if the welding is carried out correctly, the effects of the presence of this phase can be minimized [11,12]. The GMAW process has been employed in many applications, including in the oil and gas industry, due to its main characteristics, which include no spatter and welding in all positions. In the work performed by Chacón-Fernández [3], it was demonstrated that by using correct welding parameters for stainless steels, phase balance can be controlled. The most important parameters are the temperature reached, the cooling rate, and the bead geometry [13,14].

In this work, we presented the results of the study of the corrosion properties of duplex stainless steel 2205 GMAW samples by using a corrosion reactor with a hydrogen sulfide atmosphere. The novelty of this work was to perform corrosion testing on welded joints free of defects with suitable geometry in a hydrogen sulfide environment at a relatively high temperature in order to understand the metallurgical phenomena after exposure to a detrimental environment. The welding parameters were varied, and it was demonstrated that at higher heat input, ferritization was decisive for pit formation. However, when decreasing the heat input, pitting corrosion became more evident, even though this type of steel continues to be highly resistant. 

## 2. Materials and Methods

### 2.1. Materials and Welding Procedure

A UNS S32205 duplex stainless-steel plate with a thickness of 5 mm was used as the base metal (BM), and a 1.2 mm-diameter grade ER 2209 filler metal was used. The chemical compositions of both are shown in Table 1.

**Figure 1:**
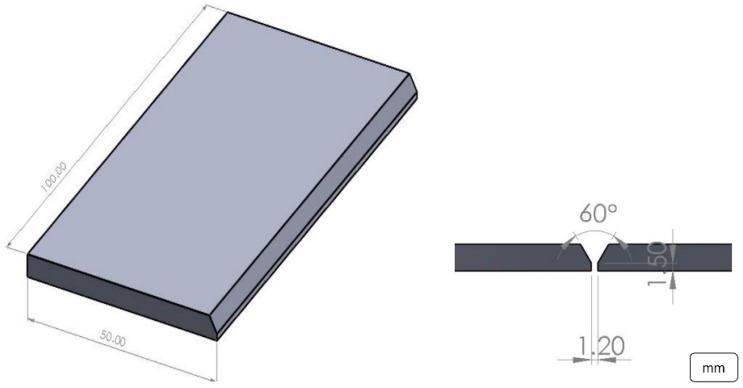
Schematic of the joint.

Single-pass welds were made using a fully automated GMAW process, using a KUKA robotic arm. The plates were machined to prepare single V-groove butt joint configurations with a 60° groove angle (see Figure 1). The GMAW spray transfer mode was performed, and a mixture of 85% Ar-15% CO_2_ at 40 ft^3^/h was used as a shielding gas. Table 2 presents the welding parameters used in this work.

### 2.2. Heat Input Calculation

The heat input was calculated according to the given Equation (1). It was assumed to have an 80% efficiency (*ƞ*)

(1) $$HI=\;\frac{\left(V\times I\right)}{S}\;\eta$$

where *HI* is the heat input in kJ/mm, *V* is the welding voltage in volts, *I* is the welding current in amperes, and *S* is the welding speed in mm/s.

### 2.3. Microstructural Characterization

For investigating the macro- and microstructural changes, the specimens were prepared using conventional metallographic methods (in accordance with ASTM.E3). The specimens were etched with Beraha’s for 10 s (3 g NH_4_F·HF + K_2_S_2_O_5_ + 25 mL HCl + 125 mL H_2_O) to distinguish different austenite phases and small precipitates such as sigma Cr_2_N, etc. In addition, Marble’s reagent (10 g CuSO_4_ + 50 mL HCl + 50 mL H_2_O) was used for 60 s to evaluate the macrostructure of the joints.

The macrostructure was inspected using a stereoscope (Nikon SMZ 745T), and the microstructure was characterized by optical microscopy (OM; Nikon Eclipse MA200) and SEM (Tescan, MIRA3) coupled with EDS. The samples before the analysis with scanning electron microscopy (TESCAN MIRA 3) were mounted in thermosetting phenolic resin (Bakelite), using graphite tape to generate better conductivity in the analysis. Subsequently, they were placed on an aluminum plate covered with graphite tape and silver paint, then introduced into the vacuum analysis chamber of the equipment using the following parameters: HV 15 kV, WD = 16 mm, view field = 27.7 mm, and secondary (SE) and backscattered (BSE) electrons for the corresponding evaluation. Atomic Force Microscopy (AFM) equipment (Nanosurf) was employed for topographic analysis of pits on a 50 µm × 50 µm surface using a Tao190Al-G cantilever in intermittent contact mode. The software used by the AFM was Nanosurf-Easyscan 2.

### 2.4. Mechanical Properties Characterization

The microhardness was measured in the transverse direction of welding across the base metal, heat-affected zone, and weld metal using a microhardness tester (Wilson Hardness Tukon 2500, Lake Bluff, IL, USA) with a load of 500 g_F_.

### 2.5. Corrosion Test

The corrosion tests were carried out using the NACE-TM0177-96 and NACE-TM0284-2011 standards as references. A particular accelerated corrosion reactor employing a synthetic seawater solution (250 g of NaCl, 25 g of CH_3_COOH, and 4725 mL of distilled water) and H_2_S gas was used. The corrosion test was carried out at 70 °C for a period of 600 h, maintaining the corrosion reactor at 15 psi of pressure during the test period. 

## 3. Results

### 3.1. Macrostructure of Welds

**Figure 2:**
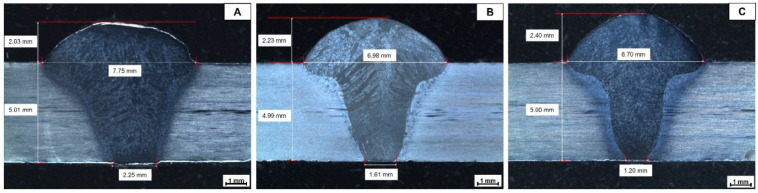
Macrostructure of the cross sections of the weld joints at different heat inputs: (**A**) high heat; (**B**) medium heat; (**C**) low heat.

Full penetration welds were obtained in each of the three input heat combinations (Figure 2). All joints present a suitable appearance, with complete penetration and no apparent distortion of the base material. In addition, no joint defects such as undercuts, porosity, or hot cracking were observed, indicating that the welding parameters and robotic process control were appropriate to avoid these types of defects. 

### 3.2. Microstructure

**Figure 3:**
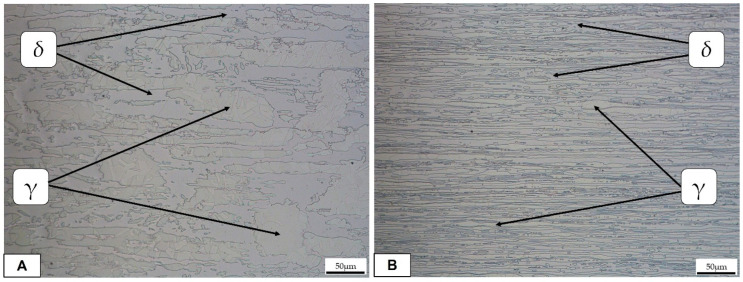
Optical micrographs (50X) of the base metal: (**A**) longitudinal direction; (**B**) transversal direction.

Figure 3 shows the typical microstructure of a duplex stainless steel (base metal), which consists of a ferritic matrix with elongated austenite islands.

**Figure 4:**
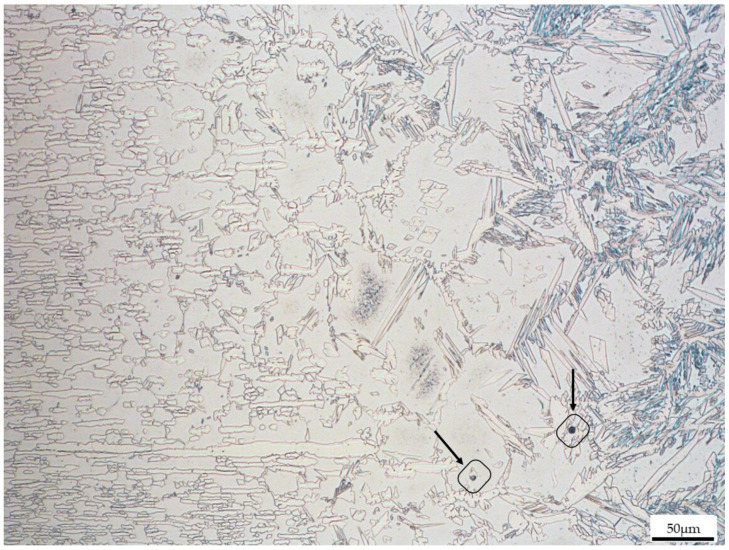
Weld zone (WZ) of Joint 1. Arrows highlight the presence of pits.

**Figure 5:**
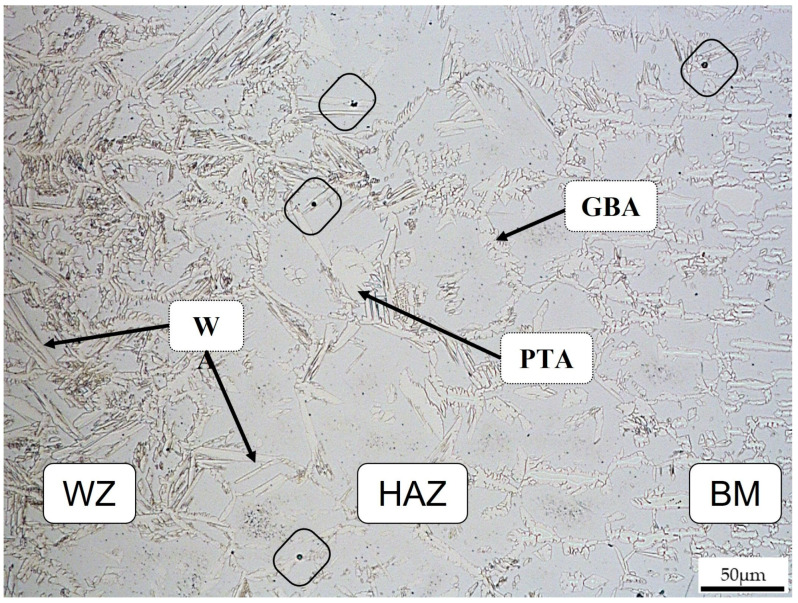
Joint 2 transition zone (WZ/HAZ/BM).

**Figure 6:**
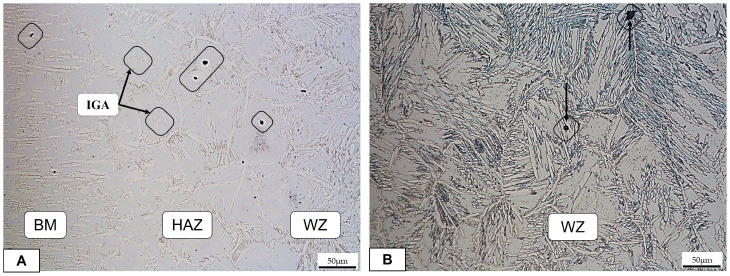
Joint 3: (**A**) transition zone BM/HAZ/WZ; (**B**) welding zone.

After 600 h of dwell time at 70 °C ± 2 °C in a saturated H_2_S atmosphere, the samples were extracted from the reactor and cut for further observation. OM was employed to observe the first preferred corrosion pits in the welded samples. The results of the microstructural analysis carried out in the different areas of the joints are shown in Figure 4, Figure 5 and Figure 6. It is important to mention that the resulting microstructure is the product of phase transformations from ferrite to austenite and vice versa, which occur during welding [12]. Both in the heat affected zone and in the welding zone of all the joints, it is possible to determine the presence of different morphologies of the austenite phase. As shown in Figure 4, Figure 5 and Figure 6, in all samples, microstructural damage is observed in the form of pitting. However, the pits were no more than 3–5 μm in diameter. In the case of sample 1, which was the one with more pitting, the preferred zone for pit nucleation and propagation was at the austenite/ferrite grain boundary, and the zone that was affected the most was the WZ. A decrease in pitting corrosion was observed in the sample with higher heat input, as shown in Figure 4. In fact, in this sample, the number of pits was fewer than in the other samples, but the pit size was larger (the pits resulted evidenced by arrows in the figure). 

The main microstructural features present in these samples include the grain boundary austenite (GBA), which is indicated in Figure 5. In the same figure, Widmanstätten austenite (WA) and partially transformed austenite (PTA) were also achieved, as observed in [4,5]. Pitting corrosion (evidenced by black squares) is present in the HAZ in the WA phase at the grain boundary and in the BM at the ferrite/austenite interface.

In Figure 6, the sample with a lower heat input resulted in more damage in all three zones. The preferential corrosion was, in this case, in the austenite phase. In fact, the ferrite/austenite boundaries for the transformed austenite phases were preferential sites for several pits. In Figure 6A, the ferrite matrix can be seen within a pitting corrosion in correspondence to the intragranular austenite (IGA), as confirmed in [15]. No visible cracks during OM observation were detected.

**Figure 7:**
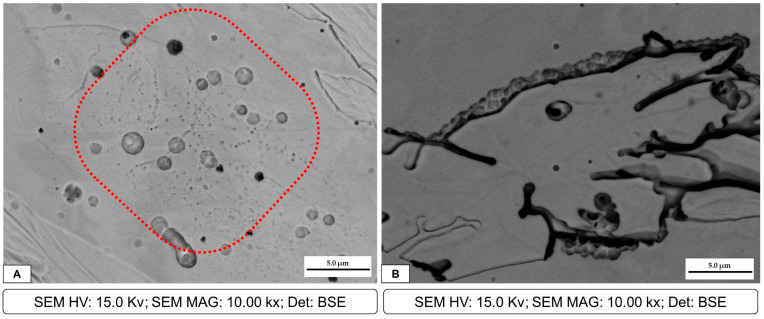
Joint 3: (**A**) pits at the IGA phase in the ferrite matrix, highlighted by the red curve; (**B**) cracks in the austenite phase and pits in the austenite/ferrite grain boundary.

**Figure 8:**
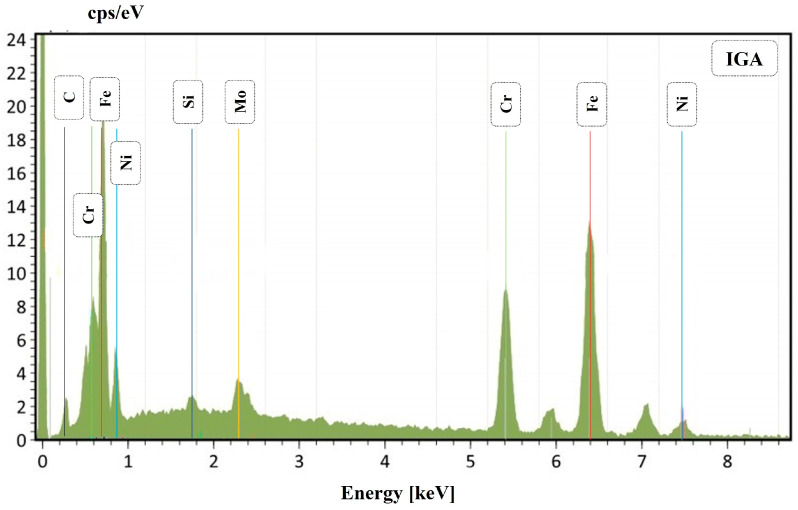
Joint 3: EDS for the IGA phase.

After OM observation, all the samples were also inspected at SEM; however, the sample that had more corrosion products was welding 3, which has the lower heat input. Two different corrosion mechanisms were detected in the welded samples. The first one is presented in the SEM micrographs of the corrosion products in Figure 7, where corrosion pits are clearly observable. These pits were also detected by OM observation, as previously reported. Figure 7A represents sample 3, which is the one with more damage. In particular, several pits were detected in the ferrite matrix. In Figure 7B, a large number of small pits can be observed at the austenite/ferrite phase boundary, outlining the austenite phase. The presence of these pits can favor crack formation and propagation. An EDS analysis of the zone highlighted in red in Figure 7A is presented in Figure 8. The IGA phase was selected in order to detect semi-quantitative differences in chemical composition. The total area for the EDS analysis was approximately 400 μm^2^ and 100 μm^2^ for Figure 8 and Figure 10, respectively. 

**Figure 9:**
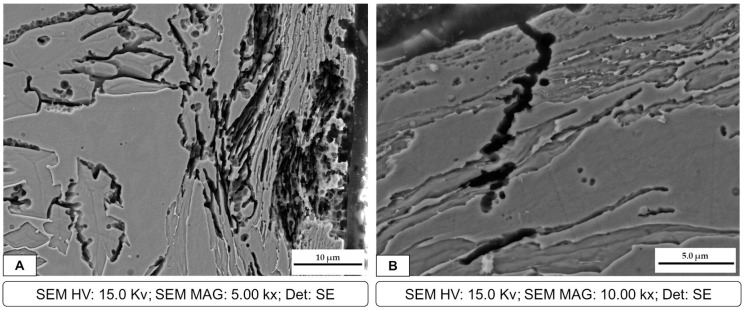
Joint 3: (**A**) cracks formed in WA and IGA phases; (**B**) crack propagation in the HAZ/WZ.

The second corrosion mechanism detected, also introduced in Figure 7, was the cracking formation. As presented in the SEM image in Figure 9A, extensive cracking through the austenite phase and at the austenite/ferrite grain boundary can be observed. However, in Figure 9B, it is highlighted that the crack propagation was through two phases, mainly at Widmanstätten austenite and in preferential zones for the IGA formation, which can be ideal zones for localized corrosion.

**Figure 10:**
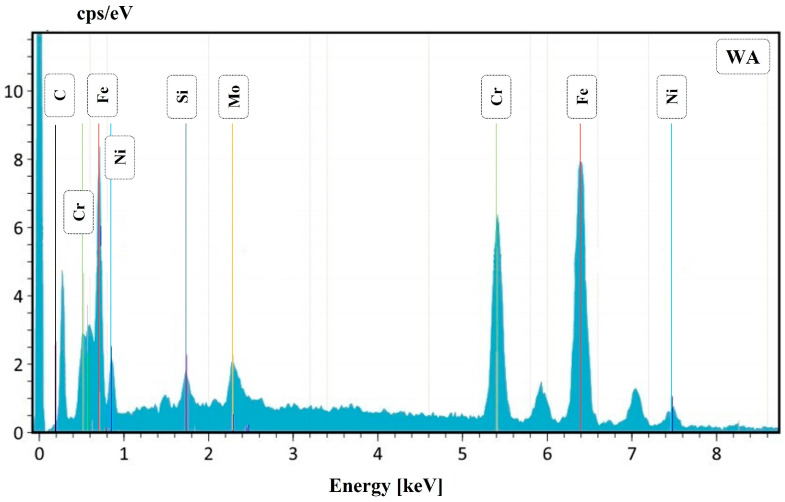
Joint 3: EDS for the WA phase.

EDS semi-quantitative analysis is shown in Table 3. It can be observed that the IGA phase has a lower Cr and Mo content compared to the GBA phase, whereas Ni and Si increase. Also, in the WA phase, a decrease in Cr and Mo can be observed. This fact can explain the previously reported behavior; in fact, in the literature, it is reported that this condition could be a trigger that ensures preference pitting for austenite [16]. 

**Figure 11:**
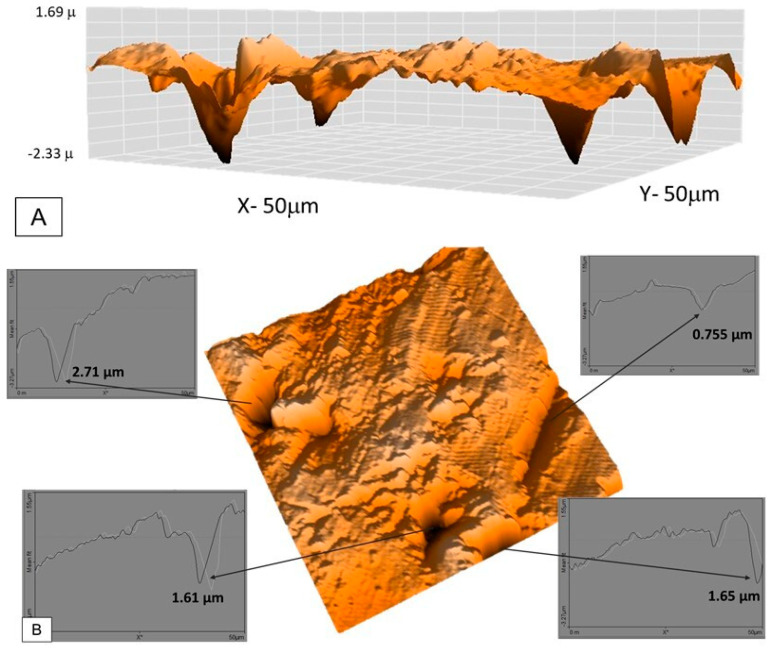
Sample 3: (**A**) pits situated in the HAZ; (**B**) pit depth penetration and crack evidence.

Sample 3 was observed using topographical analysis and an AFM, and several pits measuring 50 × 50 μm can be observed in the HAZ. Lateral topography in 3D with a scale is presented in Figure 11A. This area was selected due to the presence of pits. Figure 11B shows the penetration depth of the pits and the presence of a crack on the surface. In this specific area, the pit depth values ranged from 2.71 μm to 1.61 μm, whereas the crack depth was 0.75 μm.

### 3.3. Mechanical Properties

**Figure 12:**
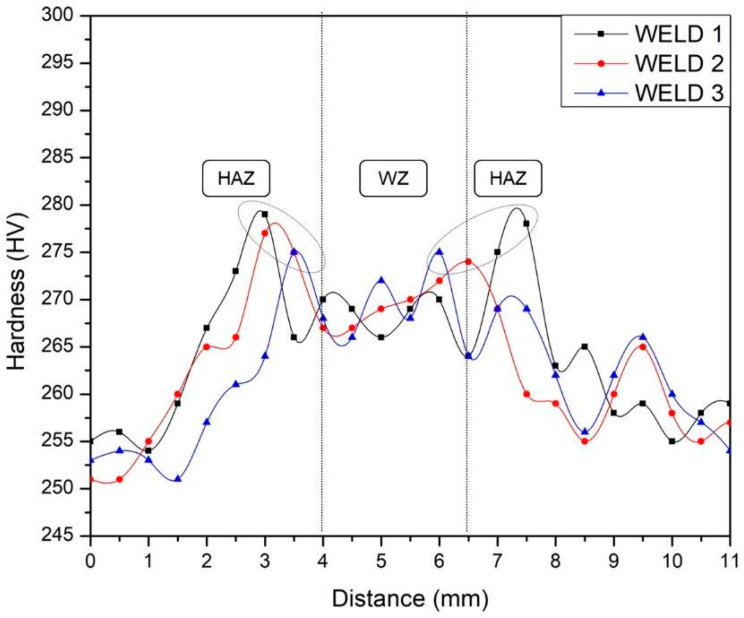
HV measurement in welds.

Microhardness measurements were taken before corrosion testing, parallel to the base plate surface, and are shown in Figure 12. The microhardness behavior in the three joints was similar. In the HAZ, an increase in hardness is observed with respect to the values of the welding zone and base metal. Previous studies have determined three factors that govern hardness in DSS: first, nitrogen content in solid solution in the austenite phase, since increasing this content promotes an increase in hardness. The precipitation of secondary phases (sigma, nitrides, carbides, etc.) is considered the second factor that generates an increase in hardness. Finally, the ferrite content and its grain size determine the hardness, and in particular, an increase in the ferrite content produces an increase in the hardness [16]. As shown in previously reported micrographs, the HAZ presents a thickened ferrite grain size with respect to the BM and WZ. In addition, its percentage in this zone compared to the base metal is higher, which leads to a substantial increase in the microhardness of the HAZ, as can be noted in Figure 12.

## 4. Discussion

Duplex stainless steels are materials with a stable microstructure at room temperature that are composed of an approximately equal percentage of ferrite and austenite, but when they are subjected to thermal cycles, they are prone to the formation of detrimental phases, which are known to be one of the reasons for corrosion resistance reduction [8]. Welding is one of the processes where thermal cycles are always present, and in these materials, the formation of detrimental phases is fundamentally related to the heat input. In this study, no formation of deleterious phases was observed in the welded samples. It was possible to observe that the size of the welding zone (WZ) increased directly in proportion to the input heat. Figure 2A shows a wider welding zone in the superior part and in the open root compared with Figure 2B,C. Also, in previous studies, it was found that both HAZ and WZ increase with increasing heat input [14,15,16]. In addition, the welding open root presents a considerable decrease in the joint with less heat input, since in this joint the decrease in the welding current leads to a reduction in the heat input, avoiding the fusion of the walls of the weld root shoulder and serving as a container for the welding electrode. The welding reinforcement varies slightly in the joints, increasing in value as the input heat decreases. This may be related to the fact that at a lower heat input, the walls of the welding groove act as containers for the base material since a complete fusion of these walls is hindered, therefore favoring an increase in the deposition of the filler material in the upper part of the weld.

Considering the microstructure, GBA formation begins once cooling of the weld starts from the untransformed austenite at the ferrite/ferrite grain boundaries [17,18,19,20,21,22,23,24]. It was expected that in both areas of the weld, the single-phase ferrite temperature was reached during heating and austenite precipitated on the mentioned grain boundaries [18]. During cooling, the amount of ferrite/ferrite grain boundary decreases, inhibiting GBA formation but allowing WA to appear at the now ferrite/austenite grain boundaries and grow towards the ferritic grain [19,20].

In addition, Figure 7 shows the presence of the IGA, which precipitates in zones enriched with Ni and N within the ferritic grain once WA formation has been completed and cooling continues. Consequently, the IGA will only form in small quantities compared to the GBA and WA [17,18,19,20,21]. As previously mentioned, during the thermal welding cycle, a small amount of austenite remains untransformed even at high temperatures, which is known as PTA and can block ferrite grain growth and inhibit the segregation of Cr and Mo [14,15]. In fact, EDS analysis showed that WA and IGA have lower quantities of Cr and Mo and are instead rich in Ni; this is due to their formation at lower temperatures and only after intergranular austenite has consumed the alloy elements [21,22]. This helps explain why these are perfect zones for pit corrosion. In addition, SEM micrographs demonstrate the formation of pitting at the austenitic grain boundaries and even the accumulation of various pitting that favors the formation of cracks throughout the entire ferritic matrix and austenite islands, depending on the chromium depletion in this zone. In some duplex stainless steels, the crack formation and propagation are in the ferrite phase. This is correlated with previous studies [8,16] where it is mentioned that the segregation of Cr and Mo in the ferrite phase promotes pitting in the austenite phase, mainly in duplex stainless steels of the first generation, while in the current ones, the high nitrogen content would generate the start of pitting in the ferrite phase. 

## 5. Conclusions

In this study, the corrosion resistance of a UNS S32205 duplex stainless steel welded by a robotic GMAW process was evaluated, and after the presentation of the experimental results, the following conclusions can be summarized:

1. The parameters employed in the robotic GMAW process were suitable for the joints, since detrimental phase formation was not detected by means of OM and SEM and the bead weld was adequate with no presence of discontinuities.
2. Three different heat inputs were obtained, which, in the higher case, promoted greater fusion on the walls of the joint. This higher heat input was also beneficial for the reduction of pitting corrosion and crack formation.
3. Two corrosion mechanisms were detected after microstructural observation: pitting corrosion and crack formation and propagation, mainly at the ferrite/austenite boundary and in some austenite phases.
4. Four types of austenite were formed: GBA, PTA, WA, and IGA. The last two mentioned were affected by Cr and Mo reduction, thus producing a remarkable decrease in the corrosion resistance of these phases.
5. No significant differences in the hardness of the welded samples can be noted. The obtained values are satisfactory for the welding technology (GMAW) and correspond to the formed phases.

## 6. Intellectual Property

The design and manufacture of the corrosion reactor are intellectually protected under the figure of industrial design with number: MX/f/2019/002399.

## Figures and Tables

**Table 1:** Chemical composition of the base metal and the filler metal (wt. %).

Material	Chemical Composition											
	C	Mn	Si	Cr	Mo	Ni	P	Co	Cu	V	W	Fe
DSS 2205	0.029	1.26	0.26	22.8	2.84	7.07	-	0.30	0.28	0.04	0.10	Bal.
ER 2209	0.02	1.57	0.14	25–29	4.00	8.0–10.0	0.10	-	0.08	0.11	-	Bal.

**Table 2:** Welding parameters used for the weld samples.

Specimen Designation	Welding Parameters				
	Current (A)	Voltage (V)	Welding Speed—WS (mm/s)	Wire Feed Speed—WFS (m/min)	Heat Input (kJ/mm)
Joint 1 (J1)	185	17.9	4.3	5.2	0.6160
Joint 2 (J2)	165	16.8	4.3	4.7	0.5157
Joint 3 (J3)	145	16.2	4.3	4.0	0.4370

Electrode extension: 9 mm. Arc length: 1.2 mm. Position: 1G.

**Table 3:** EDS analysis (wt. %) of the phase proportions.

	Elements			
Phase	Cr	Mo	Ni	Si
GBA	22.68	2.69	7.72	0.43
WA	20.90	1.03	9.97	1.9
IGA	19.77	0.80	9.1	2.3

## Data Availability

The datasets generated during and/or analyzed during the current study are available from the corresponding author upon reasonable request.
